# Abdominal Tumours and Abdominal Dropsy in Women

**Published:** 1895-09

**Authors:** 


*Abdominal Tumours and Abdominal Dropsy in Women.* By JAMES OLIVER, M.D. Pp. ix., 289. London: J. & A. CHURCHILL. 1895.

We regret that the author has departed from the time-honoured custom of commencing his book with a preface, as we might then perhaps have been informed of the object of producing a book which mentions so many diseases without a single line by which our knowledge of them is advanced. The work treats of much more than is implied by the title, and we find portions devoted to pregnancy, hernia, perityphlitis, spinal abscess, &c. In most cases the symptoms and physical signs of each disease are treated briefly; then follows a still briefer account of the treatment, in which the reader is left to take his choice; and then we have illustrative cases which fill up a large part of the book. As examples of the futility of endeavouring to get so much into the book and to so little purpose, we may mention that the operative treatment of uterine fibroids (p. 40) is summed up in ten lines; we are advised that "spontaneous rupture should, as a rule, be desired" (p. 129) in cases of pelvic peritonitis and subsequent suppuration; in about a page and a half, "perityphlitis" is dismissed without a mention of the vermiform appendix; ventral herniæ are treated with galvanism and tonics, or abdominal belts (p. 160); and it is said that in "spinal abscess," "it is probably better to allow the abscess to burst." We need surely say no more to show that the book is years behind the times, and displays an ignorance of modern surgery which is deplorable. The style of writing is rather loose and ambiguous, and we fear the British matron will be shocked by seeing in the opening paragraph that the uterus "harbours under ordinary circumstances a fecundated ovum." We feel compelled to state that we cannot recommend this book.

